# Willingness, beliefs, and barriers regarding the COVID-19 vaccine in Saudi Arabia: a multiregional cross-sectional study

**DOI:** 10.1186/s12875-021-01606-6

**Published:** 2021-12-16

**Authors:** Noura Altulahi, Shouq AlNujaim, Azzam Alabdulqader, Abdullah Alkharashi, Assaf AlMalki, Faisal AlSiari, Yara Bashawri, Sarah Alsubaie, Dayel AlShahrani, Yara AlGoraini

**Affiliations:** 1 College of Pharmacy, King Saud University, Riyadh, Saudi Arabia; 2 College of Medicine, King Saud Bin Abdul-Aziz University for Health Sciences, Riyadh, Saudi Arabia; 3 College of Medicine, Imam Abdulrahman Bin Faisal University, Dammam, Saudi Arabia; 4 Biostatistics Specialist, Biostatistics Department, King Fahad Medical City, Research Services Administration, Riyadh, Saudi Arabia; 5 Department of Pediatrics, College of Medicine, Pediatric Infectious Diseases and Infection Control, King Saud University, Riyadh, Saudi Arabia; 6 Pediatric Infectious Diseases, Pediatric Infectious Diseases Section, King Fahad Medical City, Riyadh, Saudi Arabia; 7 Pediatric Emergency Department, King Fahad Medical City, Riyadh, Saudi Arabia

**Keywords:** Willingness COVID-19 vaccine, Vaccination, Beliefs, Barriers, Saudi Arabia

## Abstract

**Background:**

The coronavirus disease 2019 (COVID-19) has spread worldwide, and the vaccine remains the ultimate cornerstone to overcoming its long-term impact. Vaccine hesitancy might obstruct the effort to achieve herd immunity and eradicate the virus. We assessed Saudi Arabian individuals’ willingness, beliefs, and barriers regarding the COVID-19 vaccine and their adherence to preventive measures during and after the pandemic.

**Methods:**

A self-administered electronic validated questionnaire was distributed among the five major regions in Saudi Arabia between November and December 2020. The questionnaire addressed the sociodemographic data, beliefs, potential barriers, parents’ acceptance of COVID-19 vaccination for their children, and adherence to protective measures during and after the pandemic.

**Results:**

Of 8,056 participants, 4,218 (52.4%) of a non-representative sample were willing to be vaccinated against COVID-19. Being a young adult, male, having less than a high school degree, being a smoker, having a chronic disease, and having a history of seasonal influenza vaccine uptake were positive predictors of COVID-19 vaccine acceptance. Hesitant participants reported concerns about vaccine side effects and safety as the main barriers to accepting the COVID-19 vaccine. Some refusers (26.1%) declared that they would reconsider vaccination only if the safety and effectiveness of the vaccine were reported by more studies.

**Conclusions:**

Our study revealed a promising willingness to accept the vaccine among the population, with positive beliefs and attitudes toward COVID-19 vaccination. However, a considerable proportion of the population was reluctant to accept the vaccine. Thus, publicly providing information about vaccine safety and implementing health education programs is crucial for increasing the public’s confidence in the vaccine.

**Supplementary Information:**

The online version contains supplementary material available at 10.1186/s12875-021-01606-6.

## Background

The coronavirus disease 2019 (COVID-19), caused by the severe acute respiratory syndrome coronavirus 2, has negatively impacted the global health capacity and economy [1]. About 80% of infected people recover without requiring hospitalization; however, one out of every five people with COVID-19 becomes critically ill [2]. Saudi Arabia (SA) has been affected by a few pandemics, including the Middle East respiratory syndrome coronavirus and the ongoing COVID-19 [3]. The COVID-19 infection quickly spread to SA, causing the deaths of thousands. According to World Health Organization report between January 3, 2020 and November 17, 2021, there have been 549,297 confirmed cases of COVID-19 with 8,818 reported deaths [4]. SA public health authorities have intervened by restricting the population in protective measures such as maintaining social distance, wearing masks, and using hand sanitizers [5]. Despite using many drugs and protective measures to prevent and treat this disease, the global demand for a vaccine is inevitable [6]. The government’s current focus is to introduce vaccines to the public to reach sufficient global immunity; however, no vaccines had been approved by the time of conducting this study [7].

Vaccination is one of the most effective ways to prevent the spread of disease and reduce complications associated with it. Vaccine acceptance and maintenance of high equitable vaccination uptake among all populations is not straightforward [8]. Several international and a few local studies have examined the willingness to accept the vaccine and the beliefs and barriers associated with it [9–20]. In Italy, a study reported a reduction in the willingness to receive the COVID-19 vaccine [9]. In addition, a study in the US found that 20% of the participants would refuse the vaccine [10]. Moreover, studies in the US and the UK found that adults showed a lower willingness and acceptance of the vaccination for their children than for themselves [11]. In contrast, a study in France showed that most participants were willing to receive the vaccine against COVID-19, similar to the report of a European study [12, 13]. A global study surveyed the potential acceptance of the COVID-19 vaccine in 19 different countries. Of the total population, 71.5% stated their willingness to accept the vaccine. However, differences in acceptance rates fluctuated from almost 90% (in China) to less than 55% (in Russia) [14].

Several factors may be associated with a higher probability of vaccine acceptance among a population, as reported by another US study, such as increasing the efficacy and decreasing the incidence of considerable adverse effects of the vaccine [15]. In China, most participants believed that doctors’ recommendations are an important factor in vaccine acceptance (80.6%), similar to another US study [16, 17]. Another study in China reported that healthcare workers (HCWs) showed a higher willingness for vaccine acceptance than the general population [18]. In SA, a study showed that only 44.7% of the participants planned to receive the vaccine, 78.9% had positive beliefs, and 79.9% were concerned about its side effects [19]. Another study in SA showed that most participants were interested in receiving vaccination [20].

The COVID-19 pandemic has spread worldwide, and the vaccine remains the key to ending its negative impact. This study aimed to assess the willingness, beliefs, and barriers regarding COVID-19 vaccination among SA’s population. Our findings play a role in advancing how the population’s beliefs and attitudes toward vaccination could change during an unusual public health crisis; this requires designing effective behavior change communication campaigns by the healthcare system such as conducting campaigns at malls, mosques, and schools, as well as in the media, to increase awareness among the public. Currently, the number of Saudi and international studies on COVID-19 vaccine hesitancy are substantial; however, our results will further enlighten the healthcare professionals and policymakers to address people’s beliefs and concerns regarding vaccinations. We also provide results on parents’ attitudes toward their own as well as their child’s vaccination, which remains an active research area. The importance of our results is not confined only to the current scenario and can be used to help combat any possible pandemics in the future. Such data will also help educate the public regarding vaccination hesitancy and acceptance by means tailored based on the public’s beliefs and concerns.

## Methods

### Study design

This was a cross-sectional study. A self-administered electronic questionnaire was distributed between November and December 2020 through social networking sites, such as WhatsApp and Twitter, targeting the Saudi population. We decided to use the self-administered electronic questionnaire to reach the population as The Saudi General Authority for Statistics reported that 88.6% of the Saudi population from the age of 15 years and older spend time on the Internet; among them, 97.34% spend their internet time on social media [21]. Prior screening questions were provided to ensure that the participants met the inclusion criteria, including age (≥ 18 years) and living in SA. The study was approved by the Institutional Review Board of King Fahad Medical City (no. IRB00010471).

### Questionnaire development

We conducted a literature review to assess and extract any related questions used in previous studies regarding the COVID-19 vaccine [14, 16–20, 22–25]. The first step of validation was face validation, which was conducted by three independent field experts. They reviewed the survey questions to capture the study objectives and ensure that they were not misleading, vague, or double-barreled. The second step was pilot testing among the population to minimize ambiguity and enhance clarity. Finally, the questionnaire was distributed in Arabic and English, and the translation was performed using a backward–forward design. The survey addressed five main sections: sociodemographic variables, participants’ beliefs about the COVID-19 vaccination, their willingness to get the vaccine, any potential barriers to the vaccination, parents’ willingness for their children to get the vaccine, and participants’ behavior during and after the pandemic.

### Sample size justification

While presuming that 50% of the Saudi adult population have an awareness of the COVID-19 pandemic and allowing 5% error on either side of the stated prevalence (or 90% power) at a 95% confidence interval (CI), the estimated sample size was derived using PASS 11 software by Cochran’s method. The estimated sample size for each of the geographical regions was 1,574.

Therefore, a population of 7,870 participants were derived by multiplying the initial target sample size by five to reflect the five geographic areas.

Our collected sample was non-representative of the Saudi population; it deviates from the national population census from the SA General Authority for Statistics [26] by age group and sex. Most of our sample were women (54.2%), while 57.7% of SA’s citizens are men. Moreover, most participants were young adults (43.8%), followed by middle-aged adults (43.2%), and older adults (13.0%); in contrast, the proportions in SA are 14.5%, 52%, and 8.9%, respectively. No other sociodemographic information such as nationality, residence, marital status, educational level, or professional background were available.

### Data management and analysis

Demographic and clinical characteristics of the study participants were reported as counts (percentage). Chi-square test was used to assess the signifcance of the association (contingency) between COVID-19 vaccine acceptance and sociodemographic variables. Factors that were potential predictors of willingness were analyzed using logistic regression analyses. Stepwise multivariate logistic regression analyses were used with factors that were significant (≤ 0.30) in the univariate analysis. All analyses were performed using SPSS version 25.0 (SPSS Inc., Chicago, IL, USA). A p-value (two-tailed) ≤ 0.05 was considered significant.

## Results

In total, 8,056 participants completed the survey (Table 1), and they were mostly young (18–28 years; 43.8%) or middle-aged (29–50 years; 43.2%) adults. More than half of the participants were women (54.2%), and most had at least a university degree (75.8%). A minority were healthcare providers (15.2%), and more than two-thirds (83%) denied having any chronic diseases. Most participants did not have any allergies (82.9%), and a small proportion of them were smokers (19.3%). Approximately half of the participants had received a seasonal influenza vaccine before (51.1%), and only a few reported that they had tested positive for COVID-19 (11.7%).

**Table 1:** Sociodemographic and Predicting factors of COVID-19 vaccine acceptance *n* = 8056

Variables	Total Population *n* = 8056 (%)	If COVID-19 vaccination is available, I will take it		
		NO (%)	YES (%)	
		*n* = 3838 (47.6%)	*n* = 4218(52.4%)	*P*-value
Age				
Young Adults (18–28 years)	3526 (43.8%)	1539 (43.6%)	1987 (56.4%)	< 0.0001****
Middle aged adults (29–50 years)	3482 (43.2%)	1744 (50.1%)	1738 (49.9%)	
Older adults (older than 50 years)	1048 (13.0%)	555 (53%)	493 (47%)	
Sex				
Male	3688 (45.8%)	1658 (45%)	2030 (55%)	< 0.0001****
Female	4368 (54.2%)	2180 (49.9%)	2188 (50.1%)	
Nationality				
Saudi	7483 (92.9%)	3577 (47.8%)	3906 (52.2%)	0.298
Non-Saudi	573 (7.1%)	261 (45.5%)	312 (54.5%)	
Residence				
Middle region	2893 (35.9%)	1422 (49.2%)	1471 (50.8%)	< 0.0001****
East region	2129 (26.4%)	1076 (50.5%)	1053 (49.5%)	
Western region	1997 (24.8%)	912 (45.7%)	1085 (54.3%)	
North region	327 (4.1%)	146 (44.6%)	181 (55.4%)	
South region	710 (8.8%)	282 (39.7%)	428 (60.3%)	
Marital status				
Single	3511 (43.6%)	1561 (44.5%)	1950 (55.5%)	< 0.0001****
Married	4266 (53.0%)	2128 (49.9%)	2138 (50.1%)	
Widowed	40 (0.5%)	22 (55%)	18 (45%)	
Divorced/separated	239 (3.0%)	127 (53.1%)	112 (46.9%)	
Education				
Less than High school	200 (2.5%)	86 (43%)	114 (57%)	< 0.0001****
High school	1747 (21.7%)	754 (43.2%)	993 (56.8%)	
University and higher	6109 (75.8%)	2998 (49.1%)	3111 (50.9%)	
Professional background				
Healthcare provider	1228 (15.2%)	599 (48.8%)	629 (51.2%)	0.189
Employed	4294 (53.3%)	2069 (48.2%)	2225 (51.8%)	
Unemployed	2534 (31.5%)	1170 (46.2%)	1364 (53.8%)	
Do you have any chronic disease?				
No	6683 (83.0%)	3227 (48.3%)	3456 (51.7%)	0.011**
Yes	1373 (17.0%)	611 (44.5%)	762 (55.5%)	
Do you have any allergy?				
No	6682 (82.9%)	3149 (47.1%)	3533 (52.9%)	0.041*
Yes	1374 (17.1%)	689 (50.1%)	685(49.8%)	
Are you a smoker?				
No	6499 (80.7%)	3190 (49.1%)	3309 (50.9%)	< 0.0001****
Yes	1557 (19.3%)	648 (41.6%)	909 (58.4%)	
Did you get the seasonal influenza vaccine before?				
No	3941 (48.9%)	2070 (52.5%)	1871 (47.5%)	< 0.0001****
Yes	4115 (51.1%)	1768 (43%)	2347 (57%)	
Have you been tested positive for COVID-19?				
No	7117 (88.3%)	3422 (48.1%)	3695 (51.9%)	0.029*
Yes	939 (11.7%)	416 (44.3%)	523 (55.7%)	
If yes, were you hospitalized for COVID-19?				
No	878 (93.5%)	398 (45.3%)	480 (54.7%)	0.018*
Yes	61 (6.5%)	18 (29.5%)	43 (70.5%)	

*Statistically significant at *p* < 0.05

** statistically significant at *p* < 0.01

*** statistically significant at *p* < 0.001

**** statistically significant at *p* < 0.0001

Of the 8,056 participants, 47.6% were not willing to receive the vaccine, while 52.4% were willing to be vaccinated against COVID-19. The proportion of those who were willing to accept the vaccine was higher among the young adults (56.4%), men (55%), those with chronic diseases (55.5%), smokers (58.4%), those with a high school degree (56.8%), those who had received seasonal influenza vaccine (57%), and those with less than a high school degree (57%; Table 1).

A logistic regression analysis of the positive predictors of COVID-19 vaccine acceptance among the participants showed that, compared to their respective counterparts, young adults (odds ratios: 1.781 [95% CI: 1.528–2.075]), male participants (odds ratio: 1.153 [95% CI: 1.047–1.269]), those who had received seasonal influenza vaccine (odds ratio: 1.508 [95% CI: 1.378–1.649]), those with comorbidities (odds ratio: 1.285 [95% CI: 1.131–1.460]), smokers (odds ratio: 1.277 [95% CI: 1.133–1.439]), those with less than a high school degree (odds ratio: 1.339 [95% CI: 1.003–1.787]), and those with a high school degree (odds ratio: 1.247 [95% CI: 1.117–1.393]) were more willing to receive the COVID-19 vaccine (Table 2).

**Table 2:** Logistic regression analysis of positive predicting factors of COVID-19 vaccine acceptance

			**95% C.I.for EXP(B)**	
**Variables**	**P-value**	**OR**	**Lower**	**Upper**
** Sex** Male: Female	0.004**	1.153	1.047	1.269
** Comorbidity** Yes: No	< 0.0001****	1.285	1.131	1.460
** Allergy** Yes: No	0.020*	0.868	0.771	0.978
** Smoking** Yes: No	< 0.0001****	1.277	1.133	1.439
** Flu vaccine** Yes: No	< 0.0001****	1.508	1.378	1.649
** Age**	< 0.0001****			
Young aged adult: Older adults	< 0.0001****	1.781	1.528	2.075
Middle aged adults: Older aged adults	0.001***	1.291	1.112	1.499
** Education**	< 0.0001****			
Less than High school: University and Higher	0.048*	1.339	1.003	1.787
High school: University and Higher	< 0.0001****	1.247	1.117	1.393

*Statistically significant at *p* < 0.05

** statistically significant at *p* < 0.01

*** statistically significant at *p* < 0.001

**** statistically significant at *p* < 0.0001

Most participants had positive beliefs regarding the safety (65.4%) and effectiveness (67.2%) of the vaccine (Table 3). Additionally, they agreed on the importance of the vaccine in eradicating COVID-19. However, approximately half of the participants feared contracting COVID-19 (49.6%), and some considered themselves at high risk of contracting the disease (23.3%). Moreover, more than half of the participants believed that they might contract COVID-19 even after being immunized (51.9%). Nonetheless, more than half were willing to receive the COVID-19 vaccine when available (52.4%; Table 3).

**Table 3:** Participants’ beliefs toward COVID-19 vaccination *n* = 8056

Variables		Count (%)
** I fear catching a COVID-19 infection**	No	4061 (50.4%)
	Yes	3995 (49.6%)
** I think I am at high risk of catching a COVID-19 infection**	No	6175 (76.7%)
	Yes	1881 (23.3%)
** I think COVID-19 vaccine is important**	No	2151 (26.7%)
	Yes	5905 (73.3%)
** I think COVID-19 vaccine, whenever available, would be safe**	No	2785 (34.6%)
	Yes	5271 (65.4%)
** I think COVID-19 vaccine, whenever available, would be effective**	No	2639 (32.8%)
	Yes	5417 (67.2%)
** I think I might get infected with COVID-19 after immunization**	No	3871 (48.1%)
	Yes	4185 (51.9%)
** I think the best way to avoid COVID-19 infection is by getting the vaccine**	No	3024 (37.5%)
	Yes	5032 (62.5%)
** If COVID-19 vaccination is available, I will take it**	No	3838 (47.6%)
	Yes	4218 (52.4%)

Table 4 shows the barriers associated with COVID-19 vaccine acceptance. Many vaccine refusers were concerned about the side effects (60.03%) and safety (40.36%). Moreover, some participants believed that developing immunity following the COVID-19 infection is better than vaccination (28.06%). Others (27.85%) believed that the vaccine was unnecessary because they were practicing protective measures such as frequent hand washing and wearing masks. Approximately 24.47% did not believe that the vaccine would stop the infection, and 15.37% believed that the vaccination is a conspiracy. Vaccine refusers declared that they would reconsider if the safety and effectiveness were reported by more studies (54.85%), it was mandated by the government (40.18%), their physicians recommended it to them (22.49%), and it was required to protect their family (22.02%). However, some still refused regardless of the situation (17.61%).

**Table 4:** Participants’ barriers associated with acceptance of COVID-19 vaccination *n* = 3838

Barriers	Vaccine refusers *n* = 3838 (47.6%)
	**Count (%)**
**I am concerned about the vaccine’s side effects**	2304 (60.03%)
**I don’t believe that the vaccine will stop the infection**	939 (24.47%)
**I don’t need the vaccine because I do all the right things: I wash my hands and wear a mask and gloves**	1069 (27.85%)
**I don’t like needles**	238 (6.20%)
**The COVID-19 vaccine is a conspiracy**	590 (15.37%)
**I don’t need the vaccine because I’m young and healthy**	350 (9.12%)
**I believe in natural or traditional remedies**	253 (6.59%)
**I think COVID-19 vaccine may not be safe**	1549 (40.36%)
**The best way for protection is to develop immunity following COVID-19 infection**	1077 (28.06%)
**I am against vaccination in general**	547 (14.25%)
**I had COVID-19 already so I don't need the vaccine**	196 (5.11%)
**Other**	18 (0.47%)
**Options to encourage future COVID-19 vaccination:**	
**If my physician recommended it to me**	863 (22.49%)
**If it was mandatory by my Job**	830 (21.63%)
**If it was compulsory by the government (MOH)**	1542 (40.18%)
**If my family or friends got vaccinated**	433 (11.28%)
**If I know that more studies showed that the vaccine is safe and effective**	2105 (54.85%)
**If there is a way other than injection**	142 (3.70%)
**For family protection**	845 (22.02%)
**I would not take it in any situation**	676 (17.61%)

Of the 8,056 participants, 42% had children aged < 18 years. Half of the parents were willing to accept vaccination for their children (51.67%). The most common reason for refusing was their lack of belief in the efficacy of the vaccine (65.44%), followed by believing that developing immunity following COVID-19 infection was better than getting the vaccine (34.07%) and concerns about the side effects (31.43%).

Most participants generally showed good compliance regarding the use of face masks (90.4%), hand washing or sanitization (82.7%), avoiding crowded places (76.1%), and maintaining social distance (72.7%) during the pandemic (98.4%); they were planning to continue these measures even after the pandemic (89%). Only 2.6% of the participants reported that they did not use any protective measures during the pandemic. Overall, 41.7% of the participants reported that they would stop wearing face masks, 77.3% would reduce regular hand washing/sanitization, 55.3% would stop avoiding crowded places, and 29.8% would stop maintaining social distance after the pandemic. Only 12.2% reported that they would not use any protective measures after the pandemic.

## Discussion

The Kingdom of SA has taken drastic measures to mitigate the burden of the COVID-19 pandemic substantially. Despite these tremendous efforts, the vaccine remains the key to ending the long-term impact of COVID-19 [27]. The acceptance and refusal of the vaccination generally vary both locally and internationally, even among individuals of the same sex, age, and class [28]. We assessed the willingness, beliefs, and barriers regarding the COVID-19 vaccination across the five central regions in SA. This highlights the degree of confidence and willingness among the population, thus elucidating the factors influencing people’s decisions to accept an emergency-use released vaccine in helping authorities better manage the pandemic.

Women’s statistical dominance has also been reported in similar international and local studies [14–20, 22–25]. Our results regarding the predominance of younger adults and university degree holders in vaccine acceptance are consistent with those of other studies [14–16, 18, 19, 24, 25, 28]. Furthermore, most participants (83%) did not have any chronic illnesses, which is consistent with studies from other Arab countries and France [19, 22, 29].

These consistencies could be because of biological, social, technological, cultural, and economic factors [14–20, 22–25]. At the time of this study, we did not refer to any local or international studies where they consider the history of allergy, hospitalization, or smoking as an element of their **s**ociodemographic. However, we believed that it would be beneficial for investigating factors associated vaccine acceptance and thus included these factors.

The rates of acceptance and hesitancy toward any vaccine differ worldwide [20]. This variation may be influenced by location, time, and contextual human behavior [8, 24, 30]. Our study demonstrated that half of the participants declared their willingness to get vaccinated (52.4%). It was reassuring that most participants were not vaccine-hesitant, consistent with the findings of other studies in the US (67%) [17], France (77.6%)]12[, UK (64%) [22], China (91.3%) [16], Europe (73.9%) [13[, SA (64.72%) [20[, and Egypt (73%) [23]. In studies from Jordan, Kuwait, and other Arab countries, the acceptance rate for COVID-19 vaccines was only 29.4% [24]. Additionally, a survey conducted in SA among 3,101 participants showed that 55.3% were hesitant to accept the COVID-19 vaccine [19]. However, a participant’s willingness to accept the vaccine does not necessarily assure their actual future uptake [14].

Few studies have shown that willingness to receive a vaccine is linked to several predictive factors and barriers, including distrust in the research; the safety, efficacy, and adverse outcomes of the vaccine; risk of being infected with the virus; a lack of knowledge about the nature of vaccine-preventable diseases; and misconceptions and misinformation related to the vaccine [9–11, 13–20, 22–25]. Our study highlighted several factors that may increase or decrease people’s willingness to receive the vaccine. Concerns over vaccine side effects was the most common barrier to vaccine acceptance among refusers, consistent with the reports from SA [19], the US [12], China [18], and Europe [13]. Another main factor and barrier found in this study was the lack of trust regarding the safety of the vaccination, which was similarly reported in previous studies from Italy, the US, and SA [9, 10, 19]. This mistrust could be because of the rapid development of vaccines.

Our study observed multiple positive predictive factors of COVID-19 vaccination acceptance. The most significant factor was a history of influenza vaccine uptake, which was consistent with previous studies in SA [27], the US [19], UK [29], and China [18]. However, the rate of influenza vaccination in SA is low, as reported by studies in the western and central regions [31, 32]. Given that COVID-19 is highly infectious with high death rates, a large proportion of the population should be vaccinated. Furthermore, being a man was another positive predictive factor of COVID-19 vaccine acceptance in this study, consistent with studies conducted in SA, the US, and China [16, 17, 19, 20]. In contrast, although women are central to decision making about family health, there was a sex gap in COVID-19 vaccine acceptance and uptake because of physical, administrative, or information accessibility issues such as myths and misinformation about the vaccine in the media]33[. Further studies should explore this possibility as these data can help future vaccination campaigns focus on women. Additionally, being a young adult was another positive predictor factor, which is consistent with another study conducted in SA [19]. Young adults may be more accepting of vaccination requirements than older adults for traveling or attending large gatherings]34[. In contrast, other studies conducted in the US [17] and SA [20] showed that older adults were more willing to get vaccinated than younger adults [17]. Moreover, having a high school degree or less was a positive predictor factor in this study, similar to a study conducted in SA [19]. Lack of knowledge about the nature of the vaccine may influence people to accept the vaccine. In contrast to other studies in the US [17] and SA [20], having a college or postgraduate degree was a positive predictive factor of vaccine acceptance; thus, further studies should explore this possibility as these data can be helpful for future vaccination campaigns.

Almost half of the participants refused to receive the COVID-19 vaccine (47.6%). Most of them stated that more research was needed to validate the vaccine’s safety and effectiveness before they could accept its efficacy, similar to reports from other studies in the US and SA [15, 19]. Consistent concerns about the COVID-19 vaccine’s side effects and effectiveness have been frequently reported in previous studies [29, 30, 35]. This finding could be explained by participants’ education level; that is, their level of knowledge may add to their hesitation.

Most participants displayed positive beliefs regarding different aspects of the COVID-19 vaccine. They acknowledged the importance of the vaccine, its safety, and effectiveness once available. A reassuring percentage of respondents recognized that the vaccine was vital to stopping the spread, as reported in SA [19, 20] and Egypt [29]. Positive beliefs are fundamental because they reflect respondents’ strong demand for the vaccine. This finding also highlights that participants realize the vital role of the vaccination, which provides a good start in achieving herd immunity. However, 51.9% believed that they might get infected with COVID-19 after immunization because the vaccine causes the disease. These results are consistent with those of other studies conducted in China, SA, Egypt, the US, and the UK [15, 16, 18–20, 23, 24].

Among HCWs in our study, approximately half (48.8%) were hesitant to receive the COVID-19 vaccine. A similar result was reported in another study in SA [19]. However, a study in China showed that most HCWs were willing to receive the COVID-19 vaccine [18]. The finding regarding HCWs’ acceptance of the COVID-19 vaccine in SA is concerning because they are at high risk of being infected with COVID-19 and play an important role in influencing individuals to be vaccinated.

Most parents were willing to accept vaccination for their children, consistent with the findings of two other studies conducted in SA addressing general guardians’ beliefs about childhood vaccination [36, 37]. However, internationally, most parents are reluctant to vaccinate their children against COVID-19. In England and the US, participants were more likely to accept a COVID-19 vaccine for themselves than for their children because of concerns about the vaccine’s safety and effectiveness [11, 38]. These studies have proposed that parents’ refusal is possibly influenced by false claims that children are naturally immune to COVID-19 or cannot contract or spread the infection [11, 38]. Furthermore, 31.43% of parents in this study were concerned about the vaccine’s side effects. This fear was also observed in the US and Italy, and it was the most common reason for avoidance of childhood vaccination [39, 40]. Considering the novelty of COVID-19 and given that future vaccines are still being developed, these concerns are understandable. Ren et al. reported that half their respondents believed that older vaccines such as polio and varicella have less risk than the new ones, supporting this point of view [41]. When further analyzing the reasons behind parents’ refusal, we found that 65.44% of the respondents were doubtful about the efficacy of the vaccine.

Although wearing a mask and maintaining a safe social distance is critical for preventing the COVID-19 spread, the Centers for Disease Control and Prevention has indicated that these measures alone will not be enough to stop the virus from spreading]42].Combining COVID-19 vaccines with these strategies will be critical in halting the pandemic. According to our findings, participants who recognized the relevance of the measures mentioned above were more eager to acquire the vaccine. Therefore, governments and health institutions should put more effort in raising public knowledge of the efficacy of these interventions. No study has investigated the use and compliance of preventive measures such as face masks, washing hands, and social distancing during the pandemic and post-pandemic. However, future studies should explore this further as it can be helpful for future vaccination campaigns.

There are substantial published studies related to COVID-19 in SA. However, few studies investigated the willingness, beliefs, and barriers for COVID-19 vaccine acceptance in SA before the release of the vaccine. Two previous studies conducted in SA [19, 20] have discussed these aspects; however, the present study explores these in depth and highlights some important clinical aspects regarding vaccine acceptance, especially at such an unusual time. The vaccine acceptance rate reported among our participants (52.4%) exceeds the acceptance rate in another similar study [19]. These differences may be because of the timing of data collection, with a sudden increase in reported confirmed cases and the increasing number of deaths related to the COVID-19 virus, leading to changes in the perception of severity among the population. Thus, we believe that these different factors may encourage the population to receive the vaccine sooner to return to ordinary life. Moreover, this study used a larger snowball sample of 8,056 people compared to previous studies’ samples of 992 [20] and 3,101 people [19]. This provided a broader insight regarding the determinants of the population’s vaccine acceptance, in addition to the reasons behind their hesitancy. Furthermore, our study investigated two interesting areas, including parents’ willingness to have their children vaccinated and post-pandemic attitude, which have not been discussed in previous studies in SA and remain an active area in the research field.

We may regard these data as factors in determining the severity of such incidents, which will help the government, policymakers, and healthcare professionals effectively address the population’s concerns and behaviors toward COVID-19 vaccination and clarify their reasons for COVID-19 vaccine hesitancy.

## Conclusion

Our study revealed a promising result of a willingness to accept the vaccine among the population, with positive beliefs and attitudes toward COVID-19 vaccination. However, a considerable number of the population were vaccine hesitant. The findings help enlighten the healthcare professionals in SA to address the population’s concerns and beliefs about vaccination in general. Thus, publicly providing information about vaccine safety and implementing health education programs is crucial for increasing the public’s confidence in the vaccine. The method of sharing vaccination information, in general, can be especially significant; thus, we recommend increasing public awareness about COVID-19 vaccination in our community. It is important to develop effective healthcare strategies to improve vaccination uptake for future generations. For example, promoting accurate COVID-19 vaccination information in campaigns at mosques, malls, and schools, as well as in the media, can be highly effective. In addition, we recommend that HCWs evaluate and increase information dissemination regarding the COVID-19 vaccine to increase knowledge and improve the population’s attitude and practice.

## Limitations

This study had some limitations. First, we conducted our study using an online questionnaire rather than face-to-face interviews owing to the curfew and social distancing restrictions during the pandemic. Therefore, some biases should be considered. Second, our study was cross-sectional in nature, limiting our ability to infer causal relationships. Third, we analyzed the willingness to accept the vaccine; however, actual vaccine uptake may be lower as our study was conducted before the COVID-19 vaccine implementation started in SA (December 18, 2020). The public’s intentions and attitudes may have changed since then. Fourth, we used a non-representative sample of the Saudi population because there were variations in the number of collected responses. More responses were obtained from specific regions as compared to others. The highest response rate was noted for the Middle region as Riyadh, one of the country’s largest cities. This could make individuals in the region more aware of and concerned about COVID-19-related issues. Other factors may have played a role, such as Internet access and regional variances in attitudes regarding the usage of social media sites. Moreover, highly educated individuals were overrepresented in this sample, which may have impacted the results. In addition, there were some translation ambiguities in Arabic, which may have changed the meaning of some items.

## Supplementary Information

Additional file 1 (DOCX 18 kb)

## Data Availability

Data that support the findings in the current study are available from the corresponding author on reasonable request.

## Abbreviations

COVID-19: Coronavirus disease 2019
SA: Saudi Arabia
HCW: Healthcare worker
CI: Confidence interval
