# Supplementary material for: Willingness, beliefs, and barriers regarding the COVID-19 vaccine in Saudi Arabia: a multiregional cross-sectional study
Source: BMC Fam Pract. 2021 Dec 16;22:247. doi: 10.1186/s12875-021-01606-6 (PMC8674018; doi:10.1186/s12875-021-01606-6)
Supplement: Supplementary file 1 — Additional file 1 (DOCX 18 kb) [file 12875_2021_1606_MOESM1_ESM.docx]

**Supplement 1: Questioner**

| First Question for Inclusion vs. Exclusion | | | |
| --- | --- | --- | --- |
| 1* | I am living in Saudi Arabia | | |
| Yes. | | No. |  |

| Theme One: Demographic Data. | | | | | | | | |
| --- | --- | --- | --- | --- | --- | --- | --- | --- |
| 1 | Age? | | | | | | | |
| 18-28 | | | 29-50 | | >50 | |  |  |
| 2 | Gender? | | | | | | | |
| Male. | | | Female. | |  | | | |
| 3 | Nationality? | | | | | | | |
| Saudi. | | | Non-Saudi. | |  | | | |
| 4 | Marital Status? | | | | | | | |
| Single. | | Married. | | Widowed. | | Divorced/separated | |  |
| 5 | State of Residence? | | | | | | | |
| Middle Region. | | East Region. | | West Region. | | North Region. | | South Region. |
| 6 | Educational Level? | | | | | | | |
| Less than high school | | High School. | | University \ post grad. | | No Formal Education. | |  |
| 7 | Your Professional background is? | | | | | | | |
| Health Care Provider. | | Employed | | Unemployed | |  |  |  |
| 8 | Do you have any chronic medical condition? (multiple answers are available) | | | | | | | |
| Yes | | No | |  |  |  |  |  |
| 9 | Do you have any allergies? (e.g. Drugs, Food, ect..) | | | | | | | |
| Yes. | | No. | |  | | | | |
| 10 | Are You a Smoker? | | | | | | | |
| Yes. | | No. | |  | | | | |
| 11 | Did you get the seasonal influenza vaccine before? | | | | | | | |
| Yes | | No | |  |  |  |  |  |
| 12 | Have you been tested positive for COVID-19? | | | | | | | |
| Yes.* | | No. | |  | | | | |
| *13 | If yes, Where you hospitalized for COVID-19? | | | | | | | |
| Yes. | | No. | |  | | | | |

| Theme Two: Beliefs Toward Covid-19, and it’s vaccine. | | | |
| --- | --- | --- | --- |
| 15 | I fear catching a COVID-19 infection. | | |
| Yes. | | No. |  |
| 16 | I think I am at high risk of catching a COVID-19 infection. | | |
| Yes. | | No. |  |
| 17 | I think COVID-19 vaccine is important? | | |
| Yes. | | No. |  |
| 18 | I think COVID-19 vaccine, whenever available, would be safe? | | |
| Yes. | | No. |  |
| 19 | I think COVID-19 vaccine, whenever available, would be effective? | | |
| Yes. | | No. |  |
| 20 | I think I might get infected with COVID-19 after immunization? | | |
| Yes. | | No. |  |
| 21 | I think the best way to avoid COVID-19 infection is by getting the vaccine? | | |
| Yes. | | No. |  |

| Theme Three: Willingness to get vaccinated, and potential barriers | | | | | |
| --- | --- | --- | --- | --- | --- |
| 22 | If COVID-19 vaccination is available, I will take it | | | | |
| Yes. | | No.* |  | | |
| *23 | If not, why? (multiple answers are available) | | | | |
| I am concerned about the vaccine’s side effects. | | I don’t believe that the vaccine will stop the infection. | I don’t need the vaccine because I do all the right things: I wash my hands and wear a mask and gloves. | I don’t like needles. | The COVID-19 vaccine is a conspiracy. |
| I don’t need the vaccine because I’m young and healthy. | | I believe in natural or traditional remedies. | I think COVID-19 vaccine may not be safe. | the best way is to let nature take its course. | I am against vaccination in general. |
| I had COVID-19 already so I don't need the vaccine | | Other…… | | | |
| 24 | Under those scenarios, I would more likely to get the COVID-19 vaccine. (multiple answers are available) | | | | |
| If my physician recommended it to me. | | If it was mandatory by my Job. | If it was compulsory by the government (MOH). | If my family or friends got vaccinated. | If I know that more studies showed that the vaccine is safe and effective. |
| I would not take it in any situation. | | If there is a way other than injection. | For family protection. |  |  |

| Theme Four: parenthood and vaccination. | | | | | |
| --- | --- | --- | --- | --- | --- |
| 25 | Are you a parent to a child who is younger than 18? | | | | |
| Yes* | | No |  | | |
| *26 | Are you going to vaccinate your children if a vaccine is available? | | | | |
| Yes | | No* |  | | |
| *27 | If no, why? (multiple answers are available) | | | | |
| I am concerned about the vaccine’s side effects. | | I don’t believe that the vaccine will stop the infection. | My child doesn’t need the vaccine because we do all the right things. we wash our hands and wear masks and gloves. | My child doesn’t like needles. | The COVID-19 vaccine is a conspiracy. |
| My child doesn’t need the vaccine because he\she is young and healthy. | | I believe in natural or traditional remedies. | I think COVID-19 vaccine may not be safe. | The best way is to let nature take its course. | I am against vaccination in general. |
| I believe only parents should be vaccinated not children | | Other…… | | | |

| Theme Five: after the pandemic. | | | | | |
| --- | --- | --- | --- | --- | --- |
| 28 | Do you use any protective measures? (multiple answers are available) | | | | |
| Face mask. | | Regular hand washing/ sanitization. | Avoiding crowded places. | Keeping distance from others. | I don’t use any protective measures. |
| 29 | Are you going to continue using any protective measures after the pandemic is over? (multiple answers are available) | | | | |
| Face mask. | | Regular hand washing/ sanitization. | Avoiding crowded places. | Keeping distance from others. | I will NOT use any protective measures. |
